# High systemic immune-inflammation index is associated with carotid plaque vulnerability: New findings based on carotid ultrasound imaging in patients with acute ischemic stroke

**DOI:** 10.3389/fneur.2022.959531

**Published:** 2022-09-01

**Authors:** Lianlian Zhang, Qi Lyu, Wenyan Zhou, Xia Li, Qinggan Ni, Shu Jiang, Guofu Shi

**Affiliations:** ^1^Department of Ultrasound, The Yancheng Clinical College of Xuzhou Medical University, The First People's Hospital of Yancheng, Yancheng, China; ^2^Department of Ultrasound, Taizhou People's Hospital, Taizhou, China; ^3^Department of General Practice Medicine, The Affiliated Hospital of Jiangsu Medical Vocational College, The Sixth Affiliated Hospital of Nantong University Yancheng Third People's Hospital, Yancheng, China; ^4^Department of Burn and Plastic Surgery, The Yancheng Clinical College of Xuzhou Medical University, The First People's Hospital of Yancheng, Yancheng, China; ^5^Department of Radiology, The Yancheng Clinical College of Xuzhou Medical University, The First People's Hospital of Yancheng, Yancheng, China; ^6^Department of Cardiovascular Medicine, The Affiliated Hospital of Jiangsu Medical Vocational College, The Sixth Affiliated Hospital of Nantong University Yancheng Third People's Hospital, Yancheng, China

**Keywords:** carotid ultrasound, systemic immune-inflammation index, acute ischemic stroke, vulnerable plaque, high-resolution magnetic resonance imaging

## Abstract

Vulnerable carotid plaque is closely related to the occurrence of Ischemic stroke. Therefore, accurate and rapid identification of the nature of carotid plaques is essential. AS is a chronic immune inflammatory process. Systemic immune-inflammation index (SII) is a novel index of immune inflammation obtained from routine whole blood cell count analysis, which comprehensively reflects the state of inflammation and immune balance in the body. This study sought to explore the relationship between SII level and carotid plaque vulnerability, plaque composition characteristics, and acute ischemic stroke (AIS) severity. A total of 131 patients diagnosed with AIS presenting with a carotid atherosclerotic plaque were enrolled in this study. Using carotid ultrasound (CDU) to assess the carotid-responsible plaque properties, we divided the patients into stable plaques group and vulnerable plaques group, and analyzed the correlation between SII levels and plaque vulnerability. And we further analyzed to evaluate the correlation between high SII levels and plaque characteristics and AIS severity. In addition, Cohen's Kappa statistics was used to detect the consistency of Carotid ultrasound (US) and cervical High-resolution magnetic resonance imaging (HRMRI) in evaluating plaque vulnerability. The findings showed that the vulnerable group had higher levels of SII compared with the stable group. The high SII group had more vulnerable plaques and a high frequency of plaque fibrous cap rupture compared with the low SII group. Logistic analysis showed that a high SII level was an independent risk factor for vulnerable plaques (odds ratio [OR] = 2.242) and plaque fibrous cap rupture (OR=3.462). The results also showed a high consistency between Carotid US and HRMRI methods in the assessment of plaque vulnerability [Cohen's kappa value was 0.89 (95% CI = 0.78–0.97)] and the level of SII was positively associated with NIHSS score (*r* = 0.473, *P* < 0.001). Our study suggests that elevated levels of SII may have adverse effects on the vulnerability of carotid plaques, especially in stroke patients with vulnerable plaques with ruptured fibrous caps, which may aggravate the severity of AIS.

## Introduction

Ischemic stroke (IS) is the most common clinical subtype of stroke. It is associated with high morbidity, disability, and mortality. Thus, it is a health burden worldwide. Atherosclerosis (AS) is a major cause of atherosclerotic ischemic stroke (AIS). Studies report that AS is a chronic immune inflammatory process associated with a variety of immune-inflammatory cells and mediators and causes instability of plaque structure (1). The risk of AS increases with an increase in atherosclerotic plaque vulnerability (2). Therefore, the nature of carotid plaque should be identified in a timely and accurate manner. It is imperative to explore immuno-inflammatory markers for early and accurate prediction of carotid plaque, thus improving the prevention and treatment of AIS. The systemic immune-inflammation index (SII) is a novel index of immune inflammation, which comprehensively reflects the state of inflammation and immune balance in the body (3, 4). SII can be obtained from routine whole blood cell count analysis.

Previous studies have extensively explored the application of SII in cardio-cerebrovascular diseases, tumors, and other diseases (3–6). However, few studies have explored the relationship between SII and vulnerability of carotid plaque and the severity of AIS. The present study sought to determine the associations between SII and plaque vulnerability and AIS severity.

## Methods

### Study population

This study was approved by the Ethics Committee of the First People's Hospital of Yancheng. All participants provided an informed consent before participating in the study. The study was a bidirectional cohort study. A total of 131 patients diagnosed with AIS presenting with carotid atherosclerotic plaque admitted to the Stroke Center of the First People's Hospital of Yancheng between June 2020 and May 2021 were enrolled in this study. CDU was used to evaluate the atherosclerotic plaque properties of the responsible vessels. Inclusion criteria: (1) patients with acute internal carotid artery system (ICA) ischemic stroke, aged ≥ 18 years; (2) time from onset to admission <3 days; (3) brain MRI and the carotid US performed within 1 week after onset of neurovascular symptoms, and conditional patients underwent cervical HRMRI. Exclusion criteria: (1) patients with intracranial arterial stenosis and other causes such as vasculitis and moyamoya disease; (2) patients with severe cardiovascular disease, hematological disorder, and hepatorenal insufficiency; (3) patients with severe infection, sepsis, malignant tumor, or autoimmune disease, and patients who were taking immunosuppressants, glucocorticoids, or cytotoxic drugs during the time of recruitment; (4) patients whose two carotid arteries (common carotid artery and internal carotid artery) had no plaque or patients without at least one carotid artery occlusion; and (6) patients with incomplete clinical baseline, laboratory examination, and imaging data (poor image quality could not be distinguished).

Clinical baseline data were collected within 24 h of admission including sex, age, hypertension, diabetes, coronary heart disease, history of stroke, history of smoking, National Institutes of Health Stroke scale (NHISS) score, and Laboratory data. Laboratory data recorded in this study included routine blood test, SII, fasting blood glucose, total cholesterol, triglyceride, high-density lipoprotein cholesterol, low-density lipoprotein cholesterol, hypersensitive C-reactive protein, homocysteine, fibrinogen, and uric acid level. SII value was defined as follows: SII = P × N/L, whereby P, N, and L indicated the peripheral blood platelet, neutrophil, and lymphocyte counts (7) at admission, respectively. Hypertension was defined as systolic blood pressure ≥ 140 mmHg and/or diastolic blood pressure ≥ 90 mmHg, and patients were undergoing treatment with antihypertensive drugs (8). Diabetes mellitus was defined as fasting blood glucose level ≥ 7.0 mmol/L or at any time and (or) glucose tolerance test 2H plasma glucose level ≥ 11.0 mmol/L (9). Coronary heart disease was defined as coronary angiography showing the left main coronary artery, left anterior descending branch, circumflex branch, right coronary artery, and main branches of any lumen diameter stenosis ≥50% (10). History of smoking was defined as smoking more than one cigarette a day for more than 1 year (11). Dyslipidemia was defined as total cholesterol >5.20 mmol/L, triglyceride >1.70 mmol/L, high-density lipoprotein cholesterol >2.00 mmol/L or <0.94 mmol/L, and low-density lipoprotein cholesterol >3.36 mmol/L (12).

### Carotid US protocol

Diagnosis in all patients was performed using a Resona8 scanner (Mindray Medical System). L14-5WE linear array probe and 3C5S convex array probe were used to extend the blood vessels from the proximal to the distal segment. Continuous cross-sectional and longitudinal scans were then performed. Bilateral common carotid arteries (CCA), carotid artery bulb (CAB), and internal carotid arteries (ICA) were explored through grayscale imaging, color flow imaging, and spectral Doppler analysis. Multi-section and multi-angle imaging were performed to identify the presence of plaques, determine the plaque size, shape, echo, integrity, and degree of vascular stenosis, as well as the peak systolic velocity, end-diastolic velocity, and resistance index (13). All images of carotid atherosclerotic plaques were saved and analyzed.

### Carotid HRMRI protocol

Patients underwent HRMRI examinations through a 3.0T MRI system (Signa Pioneer, GE Healthcare, Fairfield). Head and neck joint coil and ECG gating were selected and the standard carotid artery multi-sequence contrast imaging scheme was scanned. The 2D time of flight MR angiography (TOFMRA) imaging was performed to determine the bifurcation position of the common carotid artery. The 3DTOF MRA imaging was performed after locating the common carotid artery bifurcation, followed by rotational imaging reconstruction using maximum intensity projection (MIP). The location of the plaque was determined by combining the cross-sectional position of TOF and the reconstructed image. High-resolution target scanning of black blood sequence [including Double IR T1-weighted imaging (DIRT1WI), T2-weighted imaging (T2WI), and Proton density-weighted imaging (PDWI)] were performed to explore cervical vessels and targeted plaques. Gadolinium diethylenetriamine penta-acetic acid (Gd-DTPA) enhanced SET1WI, and Gd-DTPA were administered into the cubital vein at a concentration of 0.1 mmol/kg.

### Carotid US and HRMRI interpretation

An experienced vascular radiologist with 10-year experience evaluated the carotid plaque images. The radiologist was blinded to the clinical information and HRMRI scan. A second experienced vascular radiologist with a 10-year of experience explored the vulnerability of the plaque within a month after the initial evaluation, to determine the reproducibility of the process. Carotid US criteria for judging the nature of atherosclerotic plaque were as follows: vulnerable plaque: hypoechoic plaque, with or without irregular plaque surface shape or had incomplete fibrous cap and presence of plaque blood flow signal (ulcerative plaque); and Stable plaque: plaque was isoechoic or hyperechoic, plaque surface was regular, or the fibrous cap was intact (14–16).

Two experienced radiologists (>5 years of experience in neuroradiology) blinded to the clinical information and CDU scans independently analyzed the carotid plaque images. The two radiologists conducted all data analyses separately. The consensus of the two experts was used as the final result in case of any inconsistencies.

American Heart Association (AHA) was used as HRMRI classification criteria for the determination of atherosclerotic plaques as follows (17): vulnerable plaques: type IV-V: plaques with large necrotic fat nuclei and fibrous caps and plaques with a small amount of calcification; type VI: plaque with surface ulcers, or intra-plaque bleeding, thrombosis; and stable plaques: type II: diffuse intimal thickening or small non-calcified eccentric plaques; type VII: calcified plaque; type VIII: fibrous plaque without a fat nucleus, and with a low level of calcification. HRMRI results for the determination of plaque properties were highly consistent with plaque histopathological results; therefore, the present study used HRMRI results as the non-invasive “gold standard” for the determination of plaque properties (18).

The echogenicity of carotid plaques was divided into three groups: hypoechoic, isoechoic, and hyperechoic regarding the echogenicity of the vessel lumen and adventitia. Hypoechogenicity was defined as echogenicity similar to the vessel lumen. It looks black or almost black. Hyperechogenicity was defined as echogenicity similar to the adjacent adventitia. Isoechogenicity was defined as intermediate between hypoechogenicity and hyperechogenicity (19). Irregular plaque was defined as the plaque depth variation between 0.4 and 2 mm along the contour of the lesion (20). Carotid plaque fibrous cap rupture was defined as an arcuate line-like hyperechoic discontinuity at the surface of the plaque. An ulcerated plaque was defined as a plaque surface with depression (length and depth of the depression ≥ 2 mm) on at least two sonographically accessible surfaces and a well-defined back wall at its base. Color Doppler (CDFI) shows a “crater” - like filling defect (20). Calcification was defined as a hyperechoic area posteriorly within the plaque with acoustic-shadowing (21). Thrombus was defined as an isoechogenic material that partly or totally filled the carotid artery lumen (22).

Diffusion-weighted imaging (DWI) results were interpreted and the recent acute cerebral infarction (AIS) was evaluated according to the consensus of two neuroradiologists. American Heart Association/American Stroke Association guidelines were used for ischemic stroke diagnosis (8).

### Evaluation of the severity of acute cerebral infarction

NIHSS Stroke scale is a widely used scoring index for the determination of stroke severity in clinics. The scoring method is simple and easy and can be used to objectively and comprehensively evaluate dysfunction after stroke. The evaluation result criteria and objectivity can be used as a reliable tool to determine the severity and prognosis of stroke (23, 24). The total score is 42; a higher score denotes severe neurological impairment and consequently severe stroke. A score of 0 indicates the absence of neurological impairment symptoms, <6 score defined as mild stroke, and ≥6 score defined as moderate-severe stroke (25).

### Statistical analysis

First, CDU was used to evaluate the nature of carotid responsible plaque, and the patients were divided into stable and vulnerable groups. Then, the best cut-off value of SII to evaluate plaque vulnerability was calculated through the analysis of the receiver operating characteristic (ROC) curve, and the patients were then assigned to the high SII group [≥541.27 (10^9^/L)] and low SII group [ <541.27 (10^9^/L)] according to the cut-off value of SII. Continuous data were expressed as mean ± standard deviation or median [quartile range (IQR)]. Classified data were expressed as frequencies (percentage). The normality of continuous variable distribution was determined using the Kolmogorov–Smirnov test. Data of the two groups were compared using the chi-square test and Mann–Whitney U test. Logistic regression analysis was performed to evaluate the correlation between the carotid plaque vulnerability, the characteristics of vulnerable plaques, and SII (including parameters with significant values <0.10 in univariate analysis) using odds ratio (OR) and 95% confidence interval (CI). Cardiovascular and cerebrovascular risk factors and demographic factors were adjusted for as confounding factors during logistic regression analysis. Spearman test was used to evaluate the relationship between SII and NIHSS scores (Correlation coefficient(r) <0.4 was low correlation, 0.4 ≤ r <0.7 was medium correlation, and ≥0.7 was high correlation). CDU and HRMRI were used to determine the properties of carotid plaque and the consistency between observers (kappa ≤ 0.40 for poor consistency, 0.40–0.75 for good consistency, ≥0.75 for excellent consistency). All statistical analyses were performed using SPSS software (version 26.0, IBM company, Armonk). All tests were two-tailed, with a *p*-value threshold of 0.05 for statistical significance.

## Results

### Patient characteristics

**Figure 1:**
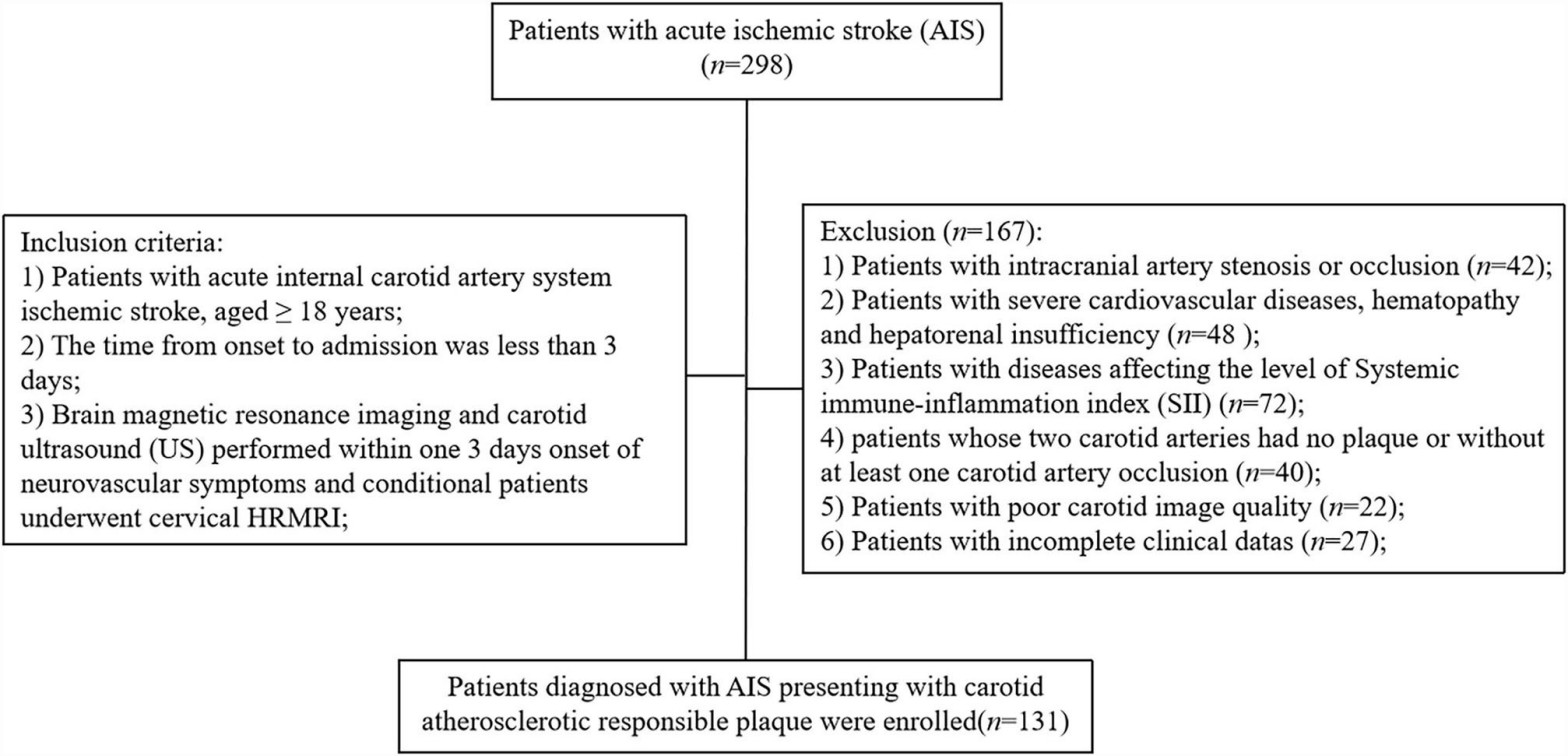
Patient inclusion and exclusion procedures.

A total of 298 patients with AIS admitted at the Stroke Center of the First People's Hospital of Yancheng between June 2020 and May 2021 were included in this study (Figure 1). Clinical and imaging examination of the 298 patients showed that 42 (14.1%) patients had intracranial artery stenosis or occlusion, 48 (16.1%) patients were diagnosed with severe cardiovascular disease, and 72 (24.2%) patients were diagnosed with diseases affecting the level of SII. Notably, 40 (13.4%) patients had at least one carotid artery occlusion, 22 (7.4%) patients presented with poor carotid image quality, and 27 (9.1%) patients were excluded due to incomplete clinical data. After exclusion and inclusion screening, a total of 131 (43.9%) patients were enrolled in the present two-way cohort study. The mean age of the 131 patients was 61.86 ± 12.37 years with 98 (74.8%) male patients. Out of the 131 patients, 57 patients (43.5%) were diagnosed with hypertension and 42 patients (32.1%) presented with diabetes; 41 (31.3%) patients reported a history of stroke and 62 (47.3%) patients were on medication (42.0% were on satin whereas 38.9% were on antiplatelet medication). Ultrasonographic findings of 131 responsible plaques and HRMRI findings of 103 (78.6%) plaques were recorded.

### Comparison of SII level between stable plaques group and vulnerable plaques group

**Figure 2:**
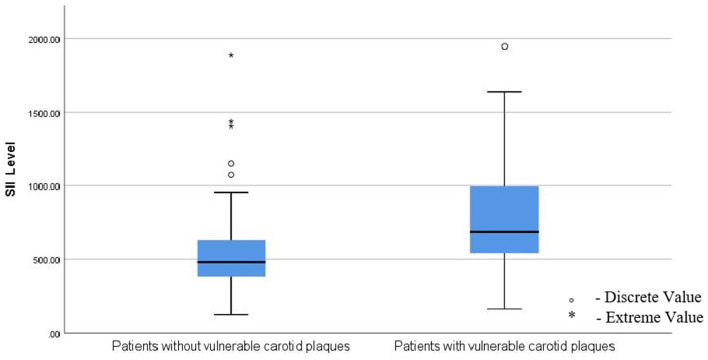
Patients in the vulnerable plaques group had higher levels of SII than those in the stable plaques group; SII, Systemic immune-inflammation index. ° means: Discrete value. * means: Extreme value.

The baseline serum SII level was 541.27 [407.23–846.27] (10^9^/L), and the patients in the vulnerable plaques group had higher SII levels than those in the stable plaques group (Figure 2). The difference was statistically significant (*P* < 0.001).

### Comparison of baseline data between high and low SII groups

**Figure 3:**
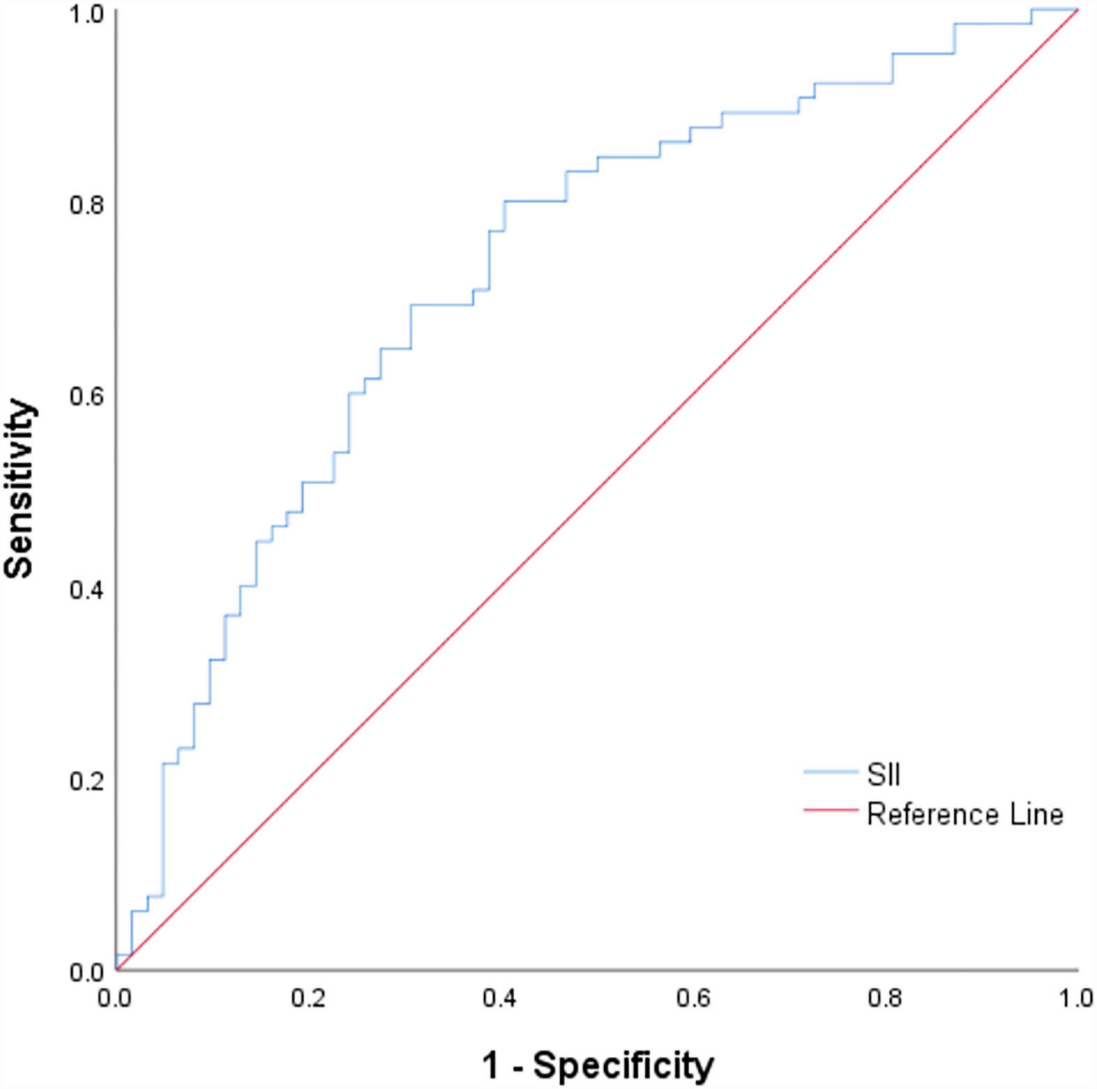
ROC curve for the SII values to predict plaque vulnerability. ROC, receiving operating characteristic curves; SII, Systemic immune-inflammation index.

The cut-off value of SII was calculated through ROC curve analysis (Figure 3). The 131 patients were assigned to two groups: SII <541.27(10^9^/L) (*n* = 52, low) and SII ≥ 541.27 (10^9^/L) (*n* = 79, high) following a method described previously (26). Baseline demographic characteristics, clinical characteristics, and laboratory results of the two groups are presented in Table 1. The results showed that admission systolic blood pressure (*P* = 0.001) and NHISS score (*P* < 0.001) were significantly different between the two groups. However, there was no significant difference in other characteristics between the two groups. Characteristics of 131 responsible plaques in the high SII group and the low SII group are presented in Table 2. The prevalence of vulnerable plaques was higher in the high SII group relative to the prevalence in the low SII group (67.1 vs. 44.2%, *P* = 0.013). The rate of rupture of plaque fibrous cap in patients with anterior circulation infarction was higher in the high SII group compared with the rate in the patients with low SII (37.9 vs. 19.2%, *p* = 0.032). The results showed no significant difference in characteristics of other plaques between the two groups.

**Table 1:** Baseline data of patients according to SII level (*N* = 131).

**Characteristics**	**SII ≥541.27(10^9^/L) (*N* = 79)**	**SII <541.27(10^9^/L) (*N* = 52)**	*P*-value^*^
Age (years)	62.26 ± 10.91	61.96 ± 12.10	0.739
Sex, male	55(83.1%)	43(86.0%)	0.614
Hypertension	37(46.8%)	17(32.7%)	0.056
Systolic blood pressure (mmHg)	143.84 ± 18.25	133.06 ± 17.21	0.001^*^
Diastolic blood pressure (mmHg)	85.04 ± 13.03	83.53 ± 11.20	0.423
Dyslipidemia	25(31.6%)	19(36.5%)	0.537
Diabetes mellitus	26(32.9%)	16(30.8%)	0.494
Coronary heart disease	30(37.9%)	18(34.6%)	0.302
History of alcohol intake	24(30.4%)	9(17.3%)	0.443
Current or former smokers	28(35.4%)	11(21.2%)	0.086
History of stroke	21(26.6%)	20(38.5%)	0.153
NHISS	11.0 [8.0–14.3]	4.0 [4.0–5.0]	<0.001^*^
**Neurological symptoms**			
Unilateral limb symptoms	38(48.1%)	29(55.8%)	0.617
Indistinct speech	21(26.6%)	14(26.9%)	0.931
Blurred vision	14(17.7%)	11(21.2%)	0.731
Dizzy	12(15.2%)	11(21.2%)	0.759
Headache	10(12.7%)	8(15.4%)	0.486
**Laboratory tests**			
Fasting blood-glucose(mmol/l)	5.74 ± 1.92	5.58 ± 1.21	0.178
Total cholesterol(mmol/l)	3.93 ± 0.94	3.99 ± 0.91	0.723
Triglycerides(mmol/l)	1.2[1.0–1.9]	1.5[1.1–1.5]	0.070
HDL-C(mmol/l)	0.97 ± 0.20	0.99 ± 0.21	0.184
LDL-C(mmol/l)	2.4[1.6–3.2]	2.3[1.9–3.1]	0.382
hs-CRP (mmol/l)	3.2[2.0–4.7]	2.7[1.5–5.9]	0.057
Uric acid(mmol/l)	324.55 ± 77.35	295.27 ± 85.15	0.269
Fibrinogen(mmol/l)	3.3[2.2–4.3]	3.1[2.2–4.7]	0.066
HCY (mmol/l)	9.8[6.7–12.4]	9.7[7.8–11.6]	0.845
**Cardiovascular medication**			
Statin treatment	35(44.3%)	20(38.5%)	0.688
Antiplatelet treatment	30(37.9%)	21(40.4%)	0.637

* means P-values indicating statistically significant. SII, systemic immune-inflammation index; NIHSS, National Institutes of Health Stroke Scale; HDL-C, high-density lipoprotein cholesterol; LDL-C, low-density lipoprotein cholesterol; hs-CRP, high-sensitive C-reactive protein; HCY, homocysteine.

**Table 2:** CDU imaging findings of carotid responsible plaques in patients with different SII levels.

**Characteristics**	**SII≥541.27(10^9^/L) (*N* = 79)**	**SII <541.27(10^9^/L) (*N* = 52)**	*P*-value^*^
Plaque presence	64(81.0%)	39(75.0%)	0.412
Vulnerable plaque	53(67.1%)	23(44.2%)	0.013^*^
**Plaque features**			
Irregular surface morphology	32(40.5%)	16(30.8%)	0.093
Ruptured fibrous cap	30(37.9%)	10(19.2%)	0.032^*^
Ulcerative plaque	13(16.5%)	9(17.3%)	0.624
LRNC or IPH prevalence	54(68.4%)	32(61.5%)	0.467
CA prevalence	37(46.8%)	30(57.7%)	0.076
Thrombus	8(10.1%)	6(11.5%)	0.732

* means P-values indicating statistically significant. CDU, color duppler ultrasound; SII, systemic immune-inflammation index; LRNC, lipid-rich necrotic core; IPH, intraplaque hemorrhage; CA, calcification.

### Relationship between the level of SII and characteristics of symptomatic carotid plaque

Univariate logistic regression and multivariate logistic analysis after adjusting for possible confounding factors showed that high SII level [odds ratio (OR) = 2.242, 95% confidence interval (CI) = 1.378–4.024, *P* = 0.023] was an independent risk factor for vulnerable plaques (Table 3). In addition, coronary heart disease [odds ratio (OR) = 4.774, 95% confidence interval (CI) = 1.337–17.049] and high SII level [odds ratio (OR) = 3.462, 95% confidence interval (CI) = 2.031–6.374] were independent risk factors for ruptured fibrous cap (Table 4).

**Table 3:** Univariate and multivariate logistic regression analysis of factors related to vulnerability plaque (*N* = 131).

	**Vulnerability plaques presence**					
	**Univariate analysis**		**Multivariate analysis**			
			**Modle1**		**Modle2**	
**Characteristics**	**OR [95% CI]**	*P*-value^*^	**OR [95% CI]**	*P*-value^*^	**OR [95% CI]**	*P*-value^*^
**Clinical parameters**						
Age (years)	1.248[0.978–3.041]	0.701	0.999[0.958–1.043]	0.989	1.001[0.957–1.047]	0.991
Sex, male	0.945[0.364–2.457]	0.908	0.760[0.175–3.311]	0.715	0.864[0.183–4.086]	0.853
Hypertension	0.796[0.395–1.600]	0.521				
Systolic blood pressure(mmHg)	1.022[1.002–1.043]	0.039^*^			1.018[0.989-1.049]	0.237
Diastolic blood pressue(mmHg)	1.016[0.987–1.045]	0.288				
Hyperlipemia	2.083[0.512–3.248]	0.852				
Diabetes mellitus	1.071[0.507–2.266]	0.857				
Coronary heart disease	2.003[0.953–4.212]	0.067				
History of alcohol intake	0.982[0.444–2.171]	0.964				
Current or former smokers	0.738[0.346–1.576]	0.433				
History of stroke	1.806[0.841–3.878]	0.129				
**Laboratory tests**						
SII≥529.87 (10^9^/L)	2.316[1.201–4.184]	0.005^*^	2.302[1.124–4.004]	0.005^*^	2.242[1.378–4.024]	0.023^*^
Fasting blood-glucose(mmol/l)	1.035[0.840–1.275]	0.746	
Total cholesterol(mmol/l)	0.666[0.446–0.994]	0.047^*^			0.687[0.182–2.590]	0.687
Triglycerides(mmol/l)	0.753[0.456–1.246]	0.270				
HDL-C(mmol/l)	0.844[0.208–3.435]	0.813				
LDL-C(mmol/l)	0.669[0.434–1.032]	0.069			1.043[0.247–4.399]	0.579
hs-CRP (mmol/l)	0.997[0.938–1.058]	0.913				
Uric acid(mmol/l)	1.000[0.996–1.004]	0.928				
Fibrinogen(mmol/l)	1.100[0.906–1.337]	0.336				
HCY (mmol/l)	1.021[0.950–1.097]	0.572				
**Cardiovascular medication**						
Statin treatment	0.745[0.370–1.501]	0.410				
Antiplatelet treatment	1.081[0.530–2.204]	0.830				

* means P-values indicating statistically significant. OR, odds ratio; CI, confidence interval; SII, systemic immune-inflammation index; HDL-C, high-density lipoprotein cholesterol; LDL-C, low-density lipoprotein cholesterol; hs-CRP, high-sensitive C-reactive protein; HCY, homocysteine. Model 1, adjusted for age and sex; model 2, adjusted for age, sex, systolic blood pressure, Total cholesterol, LDL-C.

**Table 4:** Univariate and multivariate logistic regression analysis of factors related to ruptured fibrous cap (*N* = 131).

	**Ruptured fibrous cap**					
	**Univariate analysis**		**Multivariate analysis**			
			**Modle1**		**Modle2**	
**Characteristics**	**OR [95% CI]**	*P*-value^*^	**OR [95% CI]**	*P*-value^*^	**OR [95% CI]**	*P*-value^*^
**Clinical parameters**						
Age (years)	1.001[0.968–1.035]	0.946	1.000[0.954–1.049]	0.998	1.004[0.953–1.058]	0.892
Sex, male	0.948[0.335–2.685]	0.920	0.429[0.072–2.543]	0.351	0.482[0.070–3.319]	0.459
Hypertension	1.827[0.840–3.977]	0.129				
Systolic blood pressure(mmHg)	1.024[1.003–1.046]	0.028^*^			1.015[0.981–1.051]	0.386
Diastolic blood pressure(mmHg)	1.022[0.991–1.055]	0.167				
Hyperlipemia	0.847[0.383–1.875]	0.683				
Diabetes mellitus	1.845[0.777–4.380]	0.165				
Coronary heart disease	2.611[1.196–5.700]	0.016^*^			4.774[1.337–17.049]	0.016^*^
History of alcohol intake	1.270[0.526–3.064]	0.595				
Current or former smokers	1.754[0.738–4.172]	0.203				
History of stroke	1.667[0.700–3.971]	0.249				
**Laboratory tests**						
SII≥529.87 (10^9^/L)	3.532[1.981–5.014]	0.013^*^	3.417[1.879–5.804]	0.019^*^	3.462[2.031–6.074]	0.011^*^
Fasting blood-glucose(mmol/l)	0.913[0.716–1.166]	0.467				
Total cholesterol(mmol/l)	0.545[0.343–0.866]	0.010^*^			1.137[0.199–6.480]	0.885
Triglycerides(mmol/l)	0.893[0.517–1.544]	0.686				
HDL-C(mmol/l)	0.299[0.058–1.536]	1.148				
LDL-C(mmol/l)	0.565[0.345–0.925]	0.023^*^			0.436[0.064–2.981]	0.436
hs-CRP (mmol/l)	0.985[0.921–1.053]	0.655				
Uric acid(mmol/l)	1.003[0.999–1.007]	0.144				
Fibrinogen(mmol/l)	1.051[0.878–1.257]	0.591				
HCY (mmol/l)	1.029[0.953–1.110]	0.464				
**Cardiovascular medication**						
Statin treatment	0.634[0.297–1.356]	0.240				
Antiplatelet treatment	0.572[0.266–1.231]	0.153				

* means P-values indicating statistically significant. OR, odds ratio; CI, confidence interval; SII, systemic immune-inflammation index; HDL-C, high-density lipoprotein cholesterol; LDL-C, low-density lipoprotein cholesterol; hs-CRP, high-sensitive C-reactive protein; HCY, homocysteine. Model 1, adjusted for age and sex; model 2, adjusted for age, sex, coronary heart disease, systolic blood pressure, Stroke history.

### Comparison of consistency between CDU and HRMRI imaging of the same carotid plaque

**Figure 4:**
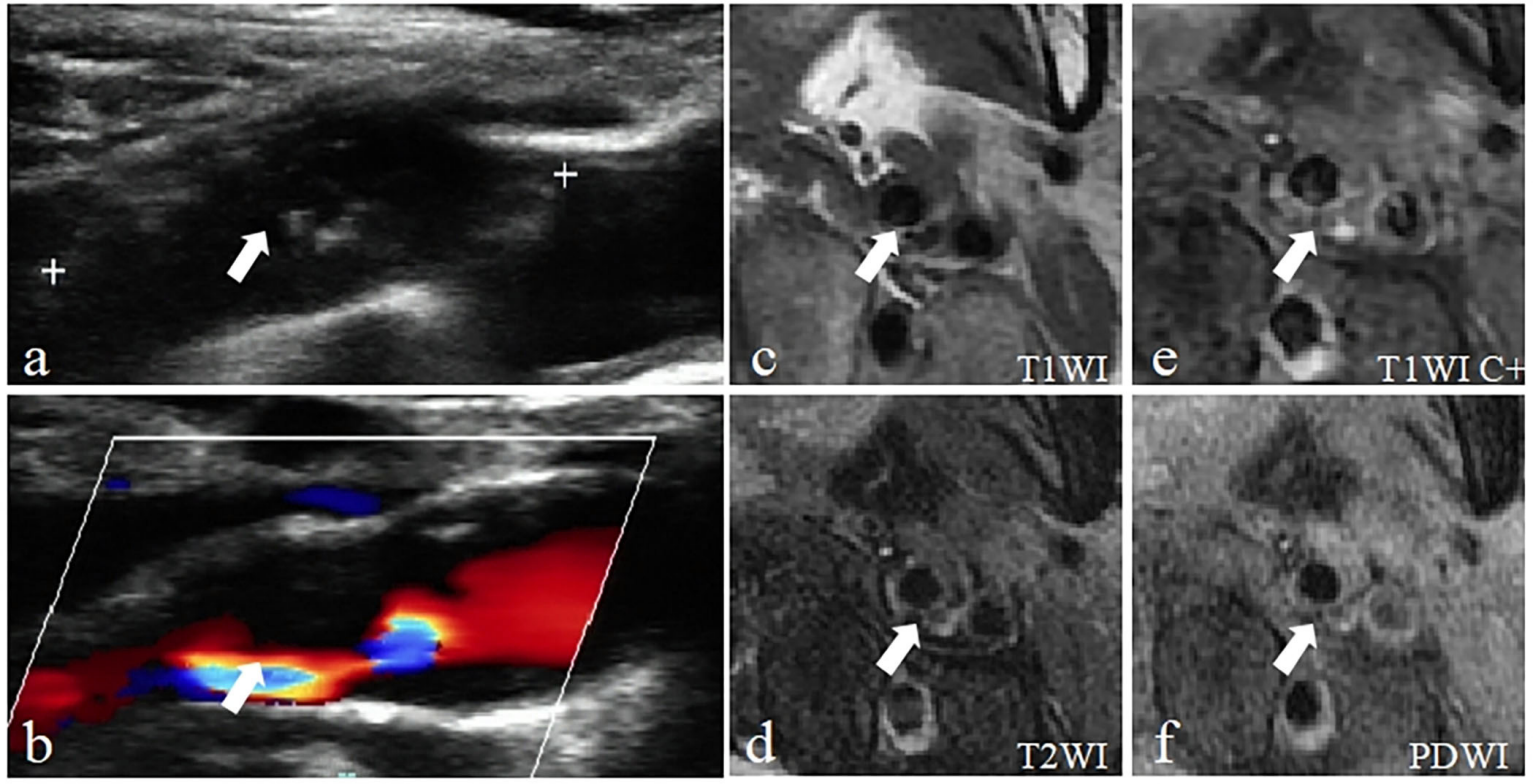
Example of a vulnerable carotid plaque of a patient using Carotid ultrasound (CDU) and High-resolution magnetic resonance imaging (HRMRI). **(a)** Gray-scale longitudinal section scan shows heterogeneous plaques in the posterolateral wall of the left carotid bulb extended to the internal carotid artery with a large hypoechoic area inside (arrow). **(b)** CDFI longitudinal section scan showed that blood flow signals were visible in the residual lumen (arrow). **(c)** The plaque is characterized by the isointensity on T1-weighted imaging (T1WI) (arrow). **(d)** T2-weighted imaging (T2WI) showing slightly high signal intensity (arrow). **(e)** Enhanced T1-weighted imaging (T1WI C+) showing regional enhancement within the AP (arrow), defined as neovascularization or inflammation, indicate the plaque is more likely to be vulnerable. **(f)** Proton density-weighted imaging (PDWI) showing slightly high signal intensity within the AP (arrow).

The consistency of the two imaging techniques was explored through a comparison of detecting characteristics of vulnerable plaques and vulnerable plaques (Figure 4, Table 2). The Cohen's kappa value of the two methods for detecting vulnerable plaques was 0.89 (95% CI = 0.78–0.97) (Table 5). The Cohen's kappa values for detecting characteristics of vulnerable plaques (irregular surface morphology, ruptured fibrous cap, ulcerative plaque, lipid-rich necrotic core (LRNC) or intraplaque hemorrhage (IPH), calcification (CA) and thrombus) were 0.77 (95% CI = 0.62–0.91), 0.78 (95% CI = 0.63–0.92), 0.80 (95% CI = 0.63–0.97), 0.86 (95% CI = 0.75–0.98), 0.70 (95% CI = 0.60–0.86), and 0.82 (95% CI = 0.62~1.00), respectively.

**Table 5:** Comparison of the consistency of CDU and HRMRI for the detection of vulnerability plaques (*N* = 76).

	**Detection of vulnerability plaques via HRMRI**		
**Item**	**Not detected**	**Detected**	**Total**
**Detection of vulnerability plaques via CDU**			
Not detected	40(52.6%)	5(6.6%)	45(59.2%)
Detected	1(1.3%)	30(39.5%)	31(40.8%)
*K*-value	0.894		
*P*-value^*^	<0.05		

* means P-values indicating statistically significant. CDU, color doppler ultrasound; HRMRI, High-resolution magnetic resonance imaging.

### Relationship between the level of SII and severity of acute cerebral infarction

**Figure 5:**
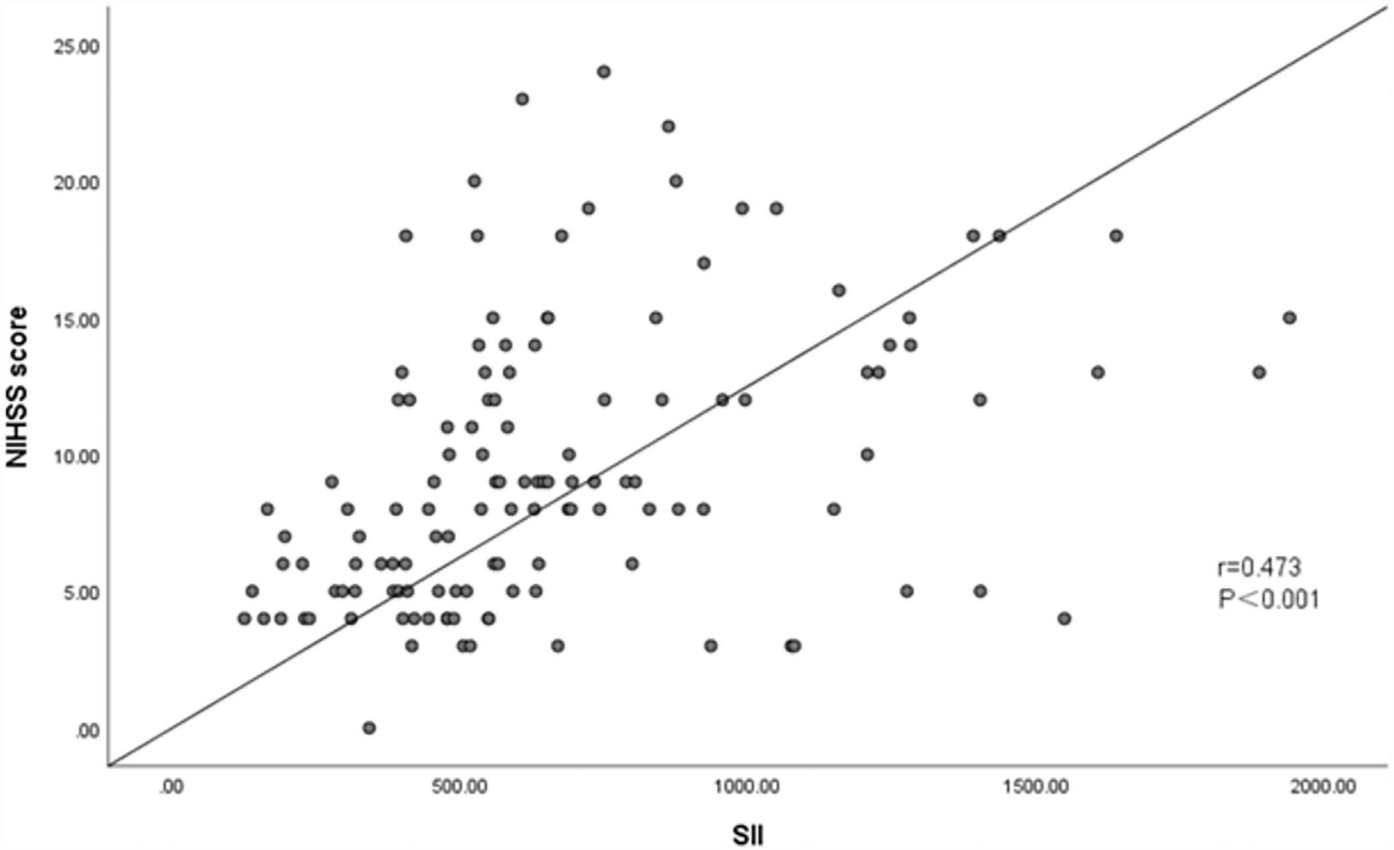
Association between SII and NHISS score. Spearman test showed that SII was positively associated with NHISS Score (*r* = 0.473, *P* < 0.001).

Spearman test showed that SII was positively associated with NIHSS score (*r* = 0.473, *P* < 0.001), suggesting a medium correlation between SII and NHISS score (Figure 5).

### Interobserver agreement

The Cohen's kappa value of carotid US for evaluation of the vulnerability of plaques by the two observers was 0.94 (95%CI = 0.92–0.98), and the Cohen's kappa values for evaluation characteristics of vulnerable plaques (irregular surface morphology, ruptured fibrous cap, ulcerative plaque, lipid-rich necrotic core (LRNC) or intraplaque hemorrhage (IPH), calcification (CA) and thrombus) by the two observers were 0.96 (95% CI = 0.92–0.98), 0.96 (95% CI = 0.91–0.97), 0.92 (95% CI = 0.89~0.95), 0.97 (95%CI = 0.94–0.99), 0.97 (95%CI = 0.94–0.0.99), and 0.97 (95% CI = 0.94–1.00), respectively.

## Discussion

Pathological features of atherosclerosis (AS) include chronic, low-grade inflammatory vascular diseases, which mainly occur in the major arteries (1). Ischemic stroke is a disease that is caused by various factors and is associated with high morbidity, disability, and mortality. Studies report that immune inflammation is involved in the occurrence, progression, and prognosis of ischemic stroke and atherosclerosis (27, 28). Several studies have explored the relationship between peripheral blood immune cell profile and AIS because the method of peripheral blood immune cell detection is simple and the results are easily obtained. Luo et al. (29), reported that neutrophil-to-lymphocyte ratio (NLR) is associated with poor early prognosis in patients with AIS or transient ischemic attack (TIA). In addition, other studies report that the platelet-to-lymphocyte ratio (PNR) is a potential independent protective factor for predicting the prognosis of AIS (30). Systemic immune-inflammation index (SII) is a novel systemic immune-inflammation index obtained through the sum of lymphocyte count (L), neutrophil count (N), and platelet count (P). SII partially represents the balance of inflammation and immunity in the body. An increase in SII indicates that inflammatory response is enhanced and the immune response is weakened. This value was initially used in predicting the prognosis of various tumor diseases (7, 31). The present study sought to investigate the relationship between the level of SII and the vulnerability of carotid plaque and the severity of AIS.

Previous studies indicate that neutrophils play a key role in the inflammatory response associated with atherosclerosis (32–34). Neutrophils secrete high levels of inflammatory mediators, chemoattractants, and oxygen-free radicals to induce endothelial cell injury and subsequent tissue ischemia. Activation of monocytes and their transformation into lipid-rich macrophages is the key process resulting in atherosclerotic lesion formation (35). On the contrary, lymphocytes play a regulatory role. Moreover, platelets play a central role in thrombosis and are correlated with the prognosis of cardio-cerebrovascular diseases (36). These findings imply that SII is associated with plaque vulnerability. The results of the present study showed that high levels of SII were an independent risk factor for cervical vulnerable plaques in patients with acute anterior circulation stroke. In addition, the findings indicated that a high level of SII was an independent risk factor for fibrous cap rupture of vulnerable plaques. The possible mechanism is that in the early stage of plaque formation which comprises the fatty streak stage and fibrous plaque stage, the plaque is relatively stable with the deposition of lipids. As a result, it aggravates ischemia and hypoxia of the lesion site. The surface of atherosclerotic plaque is gradually covered by lipids with the development of atherosclerotic plaque during hypoxia. Toxins and inflammatory mediators stimulate the deposition of membrane complexes owing to a lack of adventitia formation, which further reduces the diffusion of oxygen to the vessel wall, leading to the release of vascular endothelial growth factor (VEGF). The release of these vascular growth factors promotes sprouting migration and proliferation of the original microvascular endothelial cells and induces matrix remodeling and other changes such as budding to form new capillaries. Neovascularization in plaque is characterized by high permeability. Moreover, abundant nutrient vessels increase the infiltration of lipid and inflammatory cells into carotid atherosclerotic plaque, which is the molecular channel for inflammatory cells to enter plaques. The formation of nutrient vessels further accelerates the deposition of blood lipids in plaques, aggravates infiltration of inflammatory cells, and formation of cytokines. Production of various cytokines gradually weakens the fibrous cap of the plaque, leading to interruption of the continuity of the fibrous cap of the plaque and exposure to the surface.

The results of the present study indicated that a high SII level is a potential predictor of the severity of an acute ischemic stroke, which is consistent with findings from previous studies (31). Neutrophils and lymphocytes are the main immune inflammatory cells and they play an important role in inducing ischemic brain injury. The expression level of inflammatory mediators in normal brain tissue is very low. The occurrence of ischemic injury in brain tissue can induce the release of pro-inflammatory cytokines, and promote the recruitment and accumulation of inflammatory factors and immune cells. Further, ischemic injury can aggravate neurological dysfunction by destroying the blood-brain barrier and inducing a loss of adaptive immune response (37).

A variety of imaging methods are available for evaluating the vulnerability of carotid plaque, including carotid US, HRMRI, Computed Tomography (CT), intravascular ultrasound (IVUS), and optical coherence tomography (OCT). CT, IVUS, and OCT methods are invasive or radiative imaging techniques limiting their clinical application. HRMRI is a non-invasive method with high spatial resolution, high tissue resolution, and good reproducibility. HRMRI uses multi-sequence contrast-enhanced imaging to clearly display the substructure of blood vessels from adventitia to lumen. Therefore, it allows the identification of the internal components of carotid plaque and thus the nature of the carotid plaque can be determined. A previous study compared plaque pathological detection and HRMRI and the findings showed high consistency in the evaluation of plaque properties (38). In addition, HRMRI was more effective compared with pathological detection in timeliness. Therefore, HRMRI can be used as a “non-invasive gold standard” for the evaluation of vulnerable plaques. Carotid US has several advantages such as being non-invasive, inexpensive, simple operation, does not require radiation, and has a few contraindications. Carotid US has wide clinical application owing to these characteristics and has become the first choice method for imaging examination. Carotid US provides information on distinguishing “vulnerable” and “stable” plaques through internal echoes and morphological characterization. The echoes of plaques vary with internal components, for instance, the lipid-rich core (LRNC) is hypoechoic (39) due to the high level of fat composition. The echo of intra-plaque hemorrhage (IPH) varies with the amount of oxygenated hemoglobin resulting from varying bleeding periods, and maybe sometimes similar to that of a fat nucleus (39, 40). Notably, hypoechoic (LRNC and IPH) may overlap on carotid US sonograms, making it difficult to distinguish them, and they have low sensitivity and specificity; therefore, the two were combined in this study. Calcification showed a strong echo and the fibrous cap (FC) was a thin line-like hyperechoic layer between the plaque and blood. Heterogeneous hypoechoic or moderately hypoechoic plaques, plaques with or without irregular surface shape or incomplete fibrous cap, and ulcerative plaques with internal blood flow signals were defined as vulnerable plaques in the present study. Cohen's kappa value for carotid US and HRMRI methods for plaque vulnerability was 0.89, indicating a high consistency between the two techniques. This finding confirms the reliability of carotid US in evaluating plaque vulnerability. Cohen's kappa values of plaque vulnerability-related characteristics (such as LRNC or IPH, thrombus, ulcerative plaque, ruptured fibrous cap, irregular surface morphology, Ca) of carotid US and HRMRI were 0.86, 0.82, 0.80, 0.78, 0.77, and 0.70, respectively. The results show that the Cohen's kappa value of the remaining plaque characteristics was above 0.75 except for CA prevalence, indicating an excellent consistency between the two methods. The low Cohen's kappa value for calcification prevalence may be because the calcium component is mainly calcium hydroxyapatite, which gives a low signal in all HRMRI sequences due to its magnetic sensitivity and low proton density. Studies have not fully explored whether calcification components can lead to plaque instability. In addition, calcification of the plaque is not correlated with the texture of the plaque.

The study was a two-way cohort study conducted to explore the relationship between systemic immune-inflammation index (SII) levels, carotid plaque characteristics, and AIS severity in patients with acute anterior circulation stroke. Further, the consistency of HRMRI and carotid US in assessing carotid plaque vulnerability was explored in this study. Currently, only a few studies have explored the relationship among SII, the vulnerability of atherosclerotic plaques, and the severity of acute stroke. The main findings of this study were as follows: patients with acute internal carotid artery stroke with high SII levels had a higher number of vulnerable carotid plaque as shown by the carotid US compared with patients with low SII levels. Moreover, these patients had a high risk of rupturing the fibrous cap of plaque; a high SII level was an independent risk factor for vulnerable plaque and ruptured fibrous cap. In addition, the SII level was correlated with the characteristics of carotid plaque. Spearman's test showed that the SII level was a potential predictor of AIS severity. Carotid US and HRMRI techniques showed high consistency in evaluating the vulnerability of carotid plaques.

The current study had a few limitations: (1) This was a single-center study, thus further multicenter large-sample prospective follow-up studies should be conducted to verify the findings on the correlation among SII levels, carotid atherosclerotic disease progression, and the severity of acute cerebral infarction events. (2) Levels of neutrophils, platelets, and lymphocytes vary with time. In the present study, the SII level was only measured during admission, thus further analysis should be conducted using SII levels from different time points. (3) It was challenging to get the full cooperation of patients with severe symptoms due to the long period required for MRI examination.

## Conclusion

The results of this study indicate that increase in SII value is associated with adverse effects on carotid plaque lesions in stroke patients with carotid atherosclerosis. The effects are significant for patients with vulnerable plaques with ruptured fibrous caps and may aggravate the severity of AIS in the ICA region. These findings indicate that predicting carotid atherosclerotic plaque vulnerability and stroke severity by carotid US wall imaging and SII level detection can help in choosing effective treatment options for stroke patients. Further research is needed to perform for exploring their exact underlying mechanisms between SII and vulnerable plaques.

## Data availability statement

The raw data supporting the conclusions of this article will be made available by the authors, without undue reservation.

## Ethics statement

The studies involving human participants were reviewed and approved by the Ethics Committee of the First People's Hospital of Yancheng. The patients/participants provided their written informed consent to participate in this study. Written informed consent was obtained from the individual(s) for the publication of any potentially identifiable images or data included in this article.

## Author contributions

GS and SJ: guarantor of the article. LZ, QL, and WZ: conception, design, collection, and assembly of data. XL and QN: data analysis and interpretation. All authors contributed to the article and approved the submitted version.

## Funding

Thus work was funded by the General project of Jiangsu Medical Vocational College (Grant/Award Number: 20219119), Instructive Project of the Health Commission of Jiangsu Province (Grant/Award Number: 2021262), and Scientific Research Start-up Fund of Taizhou People's Hospital (Grant/Award Number: QDJJ202115).

## Conflict of interest

The authors declare that the research was conducted in the absence of any commercial or financial relationships that could be construed as a potential conflict of interest.

## Publisher's note

All claims expressed in this article are solely those of the authors and do not necessarily represent those of their affiliated organizations, or those of the publisher, the editors and the reviewers. Any product that may be evaluated in this article, or claim that may be made by its manufacturer, is not guaranteed or endorsed by the publisher.

## Glossary

AHA, American Heart Association; AIS, Acute ischemic stroke; AS, Atherosclerosis; AP, Atherosclerotic plaque; AUC, Area under curve; CA, Calcification; CAB, Carotid artery bulb; CCA, Common carotid artery; CDFI, Color Doppler; CDU, Carotid Ultrasound; CI, Confidence Interval; CT, Computed Tomography; DIRT1WI, Double IR T1-weighted imaging; DWI, Diffusion-weighted imaging; FC, Fibrous cap; Gd-DTPA, Gadolinium diethylenetriamine penta-acetic acid; HCY, Homocysteine; HDL-C, High-density lipoprotein cholesterol; HRMRI, High-resolution magnetic resonance imaging; hs-CRP, High-sensitive C-reactive protein; ICA, Internal carotid artery; IPH, Intraplaque hemorrhage; IS, Ischemic stroke; IVUS, Intravascular ultrasound; L, Lymphocyte; LASSO, Least absolute shrinkage and selection operator; LDL-C, Low-density lipoprotein cholesterol; LRNC, Lipid-rich necrotic core; MIP, Maximum Intensity Projection; N, Neutrophil; NHISS, National Institutes of Health Stroke scale; NLR, Neutrophil-to-lymphocyte Ratio; OCT, Optical coherence tomography; OR, Odds Ratio; P, Platelet count; PDWI, Proton density weighted imaging; PNR, Platelet-to-lymphocyte Ratio; ROC, Receiving operating characteristic; ROI, Region of interest; SII, Systemic immune-inflammation index; T1WI T1-, weighted imaging; T2WI T2-, weighted imaging; TC, Total cholesterol; TG, Triglycerides; TIA, Transient ischemic attack; TOF MRA, Time of flight MR angiography; US, Ultrasound; VEGF, Vascular Endothelial Growth Factor.
